# Pediatric canalicular laceration repair using the Mini Monoka versus Masterka monocanalicular stent

**DOI:** 10.5935/0004-2749.20220084

**Published:** 2023

**Authors:** Mehmet Serhat Mangan, Sevil Gul Turan, Serap Yurttaser Ocak

**Affiliations:** 1 Division of Ophthalmic Plastic and Reconstructive Surgery, Sadik Eratik Eye Clinic, Haydarpasa Numune Education and Research Hospital, University of Health Sciences, Istanbul, Turkey.; 2 Division of Ophthalmic Plastic and Reconstructive Surgery, Department of Ophthalmology, Okmeydani Education and Research Hospital, University of Health Sciences, Istanbul, Turkey.

**Keywords:** Eye injuries, Lacrimal apparatus/injuries, Nasolacrimal duct, Lacerations, Stents, Microsurgery, Intubation/methods, Canalicular laceration, Child, Comparative study, Traumatismos oculares, Aparelho lacrimal/lesões, Ducto nasolacrimal, Lacerações, Stents, Microcirurgia, Intubação/ métodos, Laceração canalicular, Criança, Estudo comparativo

## Abstract

**Purpose:**

One of the most important disadvantages of using Mini Monoka stents in pediatric canalicular laceration repair is premature stent loss. In this study, we aimed to compare clinical outcomes between the use of Mini Monoka and Masterka monocanalicular stents in children and discuss the potential causes of premature stent loss.

**Methods:**

The medical records of 36 patients who underwent surgical repair of canalicular lacerations were retrospectively reviewed. Children aged <18 years who underwent canalicular laceration repair with either Mini Monoka or Masterka and had at least 6 months of follow-up after stent removal were included in the study. The patients’ demographics, mechanism of injury, type of stent used, premature stent loss, and success rate were analyzed. Success was defined as stent removal without subsequent epiphora and premature stent loss.

**Results:**

Twenty-seven children fulfilled our study criteria, and their data were included in the analyses. Mini Monoka was used in 14 patients (51.9%), whereas Masterka was used in 13 patients (48.1%). The preoperative clinical features, including age, sex, and mechanism of injury, were similar between the two groups. The mean age was 8.3 ± 5.5 years in the Mini Monoka group and 7.8 ± 5.9 years in the Masterka group (p=0.61). Three patients in the Mini Monoka group (21.4%) underwent reoperation due to premature stent loss. No premature stent loss was observed in the Masterka group. As a result, the rate of success was 78.6% in the Mini Monoka group, whereas it was 100% in the Masterka group (p=0.22).

**Conclusions:**

Even though the two groups did not show any statistically significant difference in success rate, we did not observe any premature stent loss in the Masterka group. Further studies with larger and randomized series are warranted to elaborate on these findings.

## Abstract (pt)

**Objetivo:**

Uma das desvantagens mais importantes do uso de stents Mini Monoka no reparo de lacerações canaliculares pediátricas é a perda prematura do stent. Neste estudo, objetivamos comparar os resultados clínicos dos stents monocanaliculares Mini Monoka e Masterka em crianças e discutir as possíveis causas da perda prematura do stent.

**Métodos:**

Foram incluídos nesta revisão retrospectiva 36 pacientes <18 anos de idade que se submeteram ao reparo cirúrgico de uma laceração canalicular com um stent Mini Monoka ou Masterka e tiveram pelo menos 6 meses de acompanhamento após a remoção do stent. Foram analisados os dados demográficos, o mecanismo da lesão, o tipo de stent utilizado, a ocorrência de perda prematura de stent e o sucesso da intervenção. O sucesso foi definido como a ausência de epífora após a remoção do stent, sem a perda prematura deste.

**Resultados:**

Vinte e sete pacientes preencheram os critérios do presente estudo e foram incluídos nas análises. O stent Mini Monoka foi usado em 14 pacientes (51,9%), enquanto o Masterka foi usado em 13 pacientes (48,1%). As características clínicas pré-operatórias, incluindo idade, sexo e mecanismo de lesão, foram semelhantes entre os dois grupos. A média de idade foi de 8,3 ± 5,5 anos no grupo Mini Monoka e de 7,8 ± 5,9 anos no grupo Masterka (p=0,61). Três pacientes do grupo Mini-Monoka (21,4%) tiveram que ser operados novamente por perda prematura do stent. Nenhuma perda prematura do stent foi observada no grupo Masterka. Como resultado, a taxa de sucesso foi de 78,6% no grupo Mini Monoka e de 100% no grupo Masterka (p=0,22).

**Conclusões:**

Embora nenhuma diferença estatisticamente significativa tenha sido detectada entre os dois grupos em termos de taxas de sucesso, não observamos nenhuma perda prematura de stent no grupo Masterka. São necessários mais estudos, com séries maiores e randomizadas, para chegar a maiores conclusões sobre esses achados.

## INTRODUCTION

Canalicular lacerations may occur at any age but more commonly affect children and young adults^(1)^. Several surgical techniques have been described for repairing canalicular lacerations^(2,3,4,5,6)^. Stent insertion in the lacerated canaliculus using any of these techniques can be performed by almost all surgeons^(7,8,9,10,11,12,13,14,15,16,17)^.

Bicanalicular intubation requires manipulation in the uninvolved canaliculus. Therefore, it may cause iatrogenic injury, resulting in complications, including punctal or canalicular slitting, granuloma formation, superior loop dislocation, infection, and corneal abrasion^(18)^. “Pulled” bicanalicular and monocanalicular intubations both need the stent to be retrieved from the nasal cavity and require surgical experience to prevent nasal mucosal damage, which may cause bleeding, during stent retrieval^(18)^. To minimize these risks, “pushed” monocanalicular stents (Mini Monoka and Masterka) that do not involve any additional fixation have been described^(19,20)^.

Most studies regarding canalicular laceration repair report results from both adult and pediatric patients^(7,8,9,10,11,12,13,14,15,16,17)^. Studies that included only pediatric patients are limited and incorporate “pulled” monocanalicular or bicanalicular intubation^(3,4,5,6)^. To the best of our knowledge, no study has assessed the usefulness of “pushed” monocanalicular stents in the pediatric patient setting alone. Another point of interest in canalicular laceration repair studies is premature stent loss^(7,8,9,10,11,12,13,14,18)^. It is one of the most important problems that negatively impact surgical success. Children may experience premature stent loss more frequently than adults because of eye rubbing and scratching^(8,9,10)^. However, the effect of the type of stent used on the development of this complication is yet unknown.

In this study, we aimed to compare clinical outcomes between the Mini Monoka and Masterka monocanalicular stents in the repair of canalicular lacerations in children and discuss the potential causes of premature stent loss.

## METHODS

### Study design

This study was approved by our institutional review board and was conducted in accordance with the tenets of the Declaration of Helsinki. Verbal and written informed consent was obtained from the parents or guardians of all the patients. The parents/guardians also provided consent to publish any identifiable photographs of the patients.

Pediatric patients who underwent canalicular laceration repair in two tertiary referral hospitals between December 2011 and September 2020 were retrospectively examined. Patients aged <18 years who received pushed Mini Monoka or Masterka lacrimal stent placement (FCI-Ophthalmics, Marshfield Hills, MA) for a monocanalicular laceration were included in the study. The exclusion criteria were as follows: (1) patients with bicanalicular laceration, (2) patients who underwent repair using bicanalicular or monocanalicular pulled lacrimal stents, (3) patients followed up for <6 months after stent removal, or (4) patients who underwent surgical repair 2 days after trauma.

### Patient analysis

The patients were divided into two groups according to the type of stent used in surgical repair (Mini Monoka or Masterka). They underwent preoperative and postoperative ophthalmologic evaluations. Data about age, sex, mechanism of injury, involved canaliculus, type of stent used, presence of epiphora after stent removal, and stent-related complications were obtained from the patients’ medical records. Success was defined as stent removal without subsequent epiphora and premature stent loss.

### Surgical procedures

**Figure 1:**
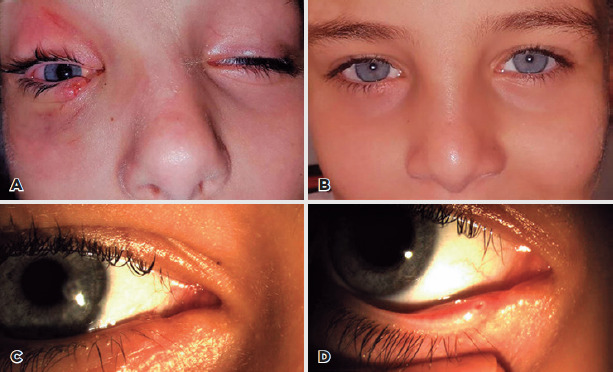
(A) A 9-year-old child with a right lower canalicular laceration caused by a sharp-force trauma. The canaliculus was stented with a 30-mm Masterka monocanalicular stent. (B) External image of the child after reconstruction. (C) Slit-lamp biomicroscopic photograph of the patient demonstrating the stent collarette apposed to the punctum. (D) The appearance of the punctum after stent removal.

The operations were performed by either of the surgeons (M.S.M. or S.G.T.). All the patients underwent monocanalicular laceration repair with either the Mini Monoka or Masterka stent under general anesthesia (Figure 1). The choice of stent (Mini Monoka or Masterka) was based on availability. The proximal torn edge of the lacerated canaliculus was explored with the aid of a surgical microscope. Assistive techniques (air, dye, or viscoelastic injection) were used when the proximal edge exploration was challenging. The punctum was gently dilated using a lacrimal punctum dilator. Then, a Mini Monoka stent was inserted in the punctum and distal lacerated canaliculus. The stent was cut short at 10 mm. After apposition of the stent collarette to the punctum, the beveled distal edge of the stent was inserted in the proximal end of the canaliculus. For Masterka stent placement, 30-mm stents were generally preferred for younger children (aged ≤10 years), whereas 35-mm stents were preferred for older children (aged >10 years). After the intubation of the proximal and distal edges of the lacerated canaliculus and nasolacrimal canal with Masterka, the stent collarette was opposed to the punctume, and then the metal guide was removed. The two edges of the lacerated canaliculus were approximated using 7-0 polyglactin sutures. The eyelid margin and skin were repaired with 6-0 or 7-0 polyglactin sutures. The stents were planned to remain in the canaliculi for 6 months after surgery in both groups.

### Statistical analyses

Statistical analyses were performed using Statistical Package for Social Sciences (SPSS) for Windows 22.0. The Mann-Whitney *U* test or Fisher exact test was used to compare the two groups. Statistical significance was defined as p values <0.05.

## RESULTS

Thirty-six patients underwent canalicular laceration repair during the study period. The data of 2 patients with bicanalicular lacerations, 3 patients who underwent repair using other stent materials, 3 patients with <6 months of follow-up after stent removal, and 1 patient who underwent surgery 4 days after the onset of trauma were excluded from the analyses.

The preoperative clinical features of the patients are presented in table 1. Mini Monoka stents were used in 14 patients (51.9%), whereas Masterka stents were preferred in 13 patients (48.1%). All the children had good anatomical repair. The dye disappearance test result after stent removal was negative and epiphora was not observed in all the cases.

**Table 1:** Clinical features of the patients with canalicular lacerations

Clinical feature	Mini Monoka	Masterka	p
Number of patients, n (%)	14 (51.9)	13 (48.1)	
Age (years)			
Mean ± SD	8.3 ± 5.5	7.8 ± 5.9	0.61
Median	6.4	4.7	
Range	1-17	1-17	
Sex, n (%)			
Male	10 (71.4)	11 (84.6)	
Female	4 (28.6)	2 (15.4)	
Mechanism of injury, n (%)			
Sharp-force trauma	8 (57.2)	9 (69.2)	
Blunt trauma	6 (42.8)	4 (30.8)	

SD= standard deviation.

Three patients in the Mini Monoka group (21.4%) underwent reoperation due to premature stent loss, whereas none of the patients in the Masterka group had premature stent loss. As a result, the success rate was 78.6% in the Mini Monoka group, whereas it was 100% in the Masterka group (p=0.22, Fisher exact test). Reoperation (new stent insertion) was performed 1 and 2 weeks after the original operation in 1 and 2 cases of the 3 patients with premature stent loss in the Mini Monoka group, respectively. All the 3 patients had inferior canaliculi lacerations. No postoperative stent migration, canaliculitis, or keratitis was observed in both groups.

## DISCUSSION

One of the most important complications of monocanalicular stent placement is premature stent loss^(7,8,9,10,11,12,13,14,18,19,20,21,22,23)^. Studies have described premature stent loss as a crucial potential problem, especially in the pediatric population, and to be correlated with surgical failure^(7,8,9,10,11,12,13,14,18)^. Premature stent loss may disrupt the healing of pericanalicular tissues^(11)^. This may cause pericanalicular or intracanalicular fibrosis and stenosis, which could reduce the surgical success rate and result in premature stent loss as one of the major causes of reoperation^(8,9,10,11,12,13,18)^. Unlike in adults, the procedure is performed under general anesthesia in children, which confers burdens from the side effects of general anesthesia, cost of the operation, and extra workload. Therefore, we thought that defining success as stent removal without subsequent epiphora and premature stent loss was more suitable. In this study with children who underwent canalicular laceration repair using either Mini Monoka or Masterka, premature stent loss was obser ved in the Mini Monoka group, for which 3 patients (21.4%) in the group had to undergo reoperation with stent reinsertion. None of the patients in the Masterka group, however, experienced premature stent loss.

Previous studies reported that the incidence rate of premature stent loss widely ranged from 3% to 43.7%^(7,8,9,10,11,12,13,14,15,16,17,18,19,20,21,22,23)^. Even though most studies about canalicular laceration repair reported the mean age of patients, they did not state the number or percentage of pediatric and adult patients^(7,8,9,10,11,12,13,14,15,16,17)^. The relatively low incidence rate of premature stent loss in previous studies about canalicular laceration repair may be related to the fact that the results of pediatric and adult patients were reported together. Even though premature stent loss may occur at any age, it is more likely to occur in children than in adults because children have greater tendencies of eye rubbing and scratching and have lower compliance^(8,9,10)^. This is supported by the point that most patients with premature stent loss in previously reported studies were, in fact, children^(8,9,10)^. We think that this may be an important factor for the reported wide range of incidence of premature stent loss. In addition, in their study on canalicular laceration repair with the Mini Monoka stent, Sendul et al.^(10)^ recommended other alternative stent materials for children because they observed premature stent loss mostly in pediatric patients. Therefore, we used Masterka as an alternative to Mini Monoka and compared their outcomes in terms of premature stent loss.

Premature stent loss may result from various mechanisms. Among pushed monocanalicular stents, unnecessarily long stents may bend by contacting the floor of the nasal space and create an upward force to dislocate the collarette^(19,20,23)^. Another possible cause may be the creation of a false passage during intubation, which may also unseat the stent by creating an upward force^(19,20,23)^. Another mechanism, especially more common in children than in adult patients, is the manipulation of the collarette by the patient. This is supported by the fact that premature stent loss is more common in children who underwent monocanalicular stent insertion for nasolacrimal duct obstruction than in children who underwent monocanalicular stent insertion for canalicular laceration repair^(21,22)^. We think that because children with canalicular laceration experienced a serious trauma, they are less likely to touch the affected zone, where the stent is placed, and manipulate the collarette owing to fear. In their two studies^(19,20)^ where Masterka stents were used for congenital nasolacrimal duct obstruction, Fayet et al. reported that the incidence rates of premature stent loss in the first postoperative week were 12.9%^(19)^ and 15%^(20)^, respectively. In one of the studies, Fayet et al.^(19)^ observed premature stent loss in 8 (12.9%) of 62 pediatric patients in the first postoperative week. The mean age of the 8 patients was 17.75 months. In 1, 2, and 5 children, 30-, 35-, and 40-mm Masterka stents were used, respectively. The researchers suggested that the Masterka stent should be longer than the distance between the lacrimal punctum and the nasal fossa^(19,20,23)^. This anatomical distance ranges between 20 and 30 mm in children and 30 and 40 mm in adults. Therefore, we think that by preferring 30- and 35-mm Masterka stents for all children in our study, we managed to minimize the risk of bending due to long stents and thus might have achieved better stent fixation.

Different from the Masterka stent, not having a metal guide, the Mini Monoka stent can increase the risk of stent bending^(12,19,20,23)^, which may in turn increase premature stent loss. The Masterka stent placement procedure is similar to probing for nasolacrimal duct obstruction. When performed by an experienced surgeon, the risk of intubating the nasolacrimal duct is higher, and the risk of false passage creation is smaller^(12,19,20,23)^. This may enable better fixation and lower the risk of extrusion. However, because Mini Monoka stents do not include a metal guide and are more difficult to implant in the canaliculus, fixation as good as in Masterka may not be achieved, which may augment the risk of extrusion. Moreover, an inappropriate stent length may cause the stent to remain or bend in the lacrimal sac, increasing the pressure and, in turn, the risk of protrusion because the Mini Monoka stent has a higher risk of bending than the Masterka stent^(24,25)^. Therefore, some authors recommend shortening the Mini Monoka stent to 10 to 25 mm^(10,17,24,26)^. Kim et al.^(17)^ reported that the risk of stent-related complications may be decreased by cutting the stent during fixation when necessary. However, we think that overcutting the stent may decrease stent fixation stability and increase the risk of protrusion. Therefore, we paid attention to not cutting the Mini Monoka stent for >10 mm.

To avoid stent extrusion, protecting the integrity of the meatic ring at the punctal opening and dilating the punctum gently using punctal dilators with small-gauge instruments are also important^(11,23)^. Lin et al.^(11)^ reported that 2 of 3 patients with premature stent loss in their series were pediatric patients and that the cause of the premature stent loss in both was excessive punctal dilation. In their study, Kaufmann et al.^(21)^ performed monocanalicular intubation with Monoka stents for congenital nasolacrimal duct obstruction. They observed a 43.7% incidence rate of premature stent loss and reported that excessive dilatation of the punctum is a predisposing factor of premature stent loss^(21)^. In our study, we cared for the punctal anatomy by using the punctal dilator under a surgical microscope in all the patients and observed no punctum-related postoperative complications.

According to previous studies, the risk factors of stent migration include excessive punctum dilation^(21)^and long stent size^(10)^. Sendul et al.^(10)^ reported that long stents may migrate by increasing the pressure in the lacrimal sac and recommended cutting the stents 10 mm shorter to prevent retrograde migration. In addition, studies have reported that the risk of intracanalicular migration decreased substantially by expanding the length of the collarette from 2 mm to 4 mm^(19,27,28)^. Tavakoli et al.^(12)^ reported no stent migration in their study, while Anastas et al.^(7)^ observed a stent migration rate of 14%. In our study, we experienced no stent migration in either of the groups.

We think that the mechanism of injury might have played an important role in achieving good anatomical repair and avoiding epiphora after stent removal in our patients. Previous studies reported that dog bite as an etiological factor is important in reducing the success rate^(5,13,29,30)^. In our study, none of the patients had dog bite as the etiology of their injuries.

The limitations of our study are its retrospective, non-randomized nature and limited sample size. Even though premature stent loss was observed in more cases in the Mini Monoka group than in the Masterka group, the difference being not statistically significant might be related to the relatively low number of cases. We can infer that the etiology of canalicular laceration and the fact that monocanalicular stent positioning is an effective procedure play important roles in the lack of a statistically significant difference, no matter which stent is used.

In conclusion, to the best of our knowledge, this is the first study to compare the outcomes of 2 pushed monocanalicular stents for canalicular laceration repair in children. Further comparative randomized studies with larger sample sizes are warranted to elaborate on these findings and thoroughly analyze the factors associated with premature stent loss.
